# Ratiometric Dissolved Oxygen Sensors Based on Ruthenium Complex Doped with Silver Nanoparticles

**DOI:** 10.3390/s17030548

**Published:** 2017-03-09

**Authors:** Zike Jiang, Xinsheng Yu, Shikui Zhai, Yingyan Hao

**Affiliations:** Key Lab of Submarine Geosciences and Prospecting Techniques, Ministry of Education, College of Marine Geosciences, Ocean University of China, Qingdao 266100, China; jiangzike2011@126.com (Z.J.); zhai2000@ouc.edu.cn (S.Z.); 15634219925@163.com (Y.H.)

**Keywords:** dissolved oxygen, ratiometric, nanofiber, silver nanoparticle, ruthenium(II) dichloride complex, electrospinning

## Abstract

A ratiometric optical sensor has been developed with electrospinning processing method for dissolved oxygen measurement. The sensing film is fabricated by using silver nano-particles (Ag NPs) doped with tris(4,7-diphenyl-1,10-phenanthroline) ruthenium(II) dichloride complex (Ru(DPP)_3_Cl_2_) encapsulated in plasticized polymethyl methacrylate (PMMA). An insensitive 3-(2-benzothiazolyl)-7-(diethy lamino)-(6CI,7CI) (Coumarin6) is adopted as reference. The ratio of oxygenation is calculated at each image pixel of a 3CCD camera to quantify the oxygen concentration in aqueous environment. Compared to Ag-free film, the response time of Ag-containing films were improved from 1.5 s to 1.0 s upon switching from deoxygenated to air saturation and from 65 s to 45 s from air saturation to fully deoxygenated. The response times of the Ag-free film obtained by knifing was 2.0 s upon switching from deoxygenated to air saturation and 104 s from air saturation to fully deoxygenated. Results of the evaluation of accuracy, limit of detection, stability, and photostability are presented. An experiment measuring the spatiotemporal variation of oxygen distribution within the photosynthesis and respiration of *Chlorella vulgaris* is demonstrated. It is shown that the nanofiber-based optical sensor film could serve as a promising method for rapid oxygen monitoring in aqueous applications.

## 1. Introduction

Oxygen is essential for nearly all biological systems on Earth, and it is one of the most important for chemical and biological processes. Knowledge of the actual oxygen concentration is of particular significance in areas such as medical diagnostics, biology, biotechnology, marine biogeochemical research, and environmental analysis [1]. It has been shown that the optical oxygen optode has the superiorities over electrochemical sensors: not O_2_ consumption, without the reference electrode, insensitive to electromagnetic noise, low cost, and it can be implemented for remote sensing.

Optical oxygen sensors are based on the mechanism of quenching by molecular oxygen. The oxygen-sensing dye is excited and then emits light of which the intensity, decay time, or wavelength is dependent on the oxygen concentration. Several dyes have been used for luminescence-based oxygen sensors, such as polycyclic aromatic hydrocarbons [2], quinoline, pyrenebutyricacid [3], transition metal–ligand complexes of palladium and iridium [4,5], osmium [6], rhenium [7,8], ruthenium [9,10,11,12,13,14,15], platinum [16,17], metalloporphyrins, and polypyridine complexes [18]. The luminescence 61-(p-hydroxyphenyl methano) fullerene has also been reported for oxygen sensing by covalently immobilization [19]. In applications for sensing dissolved oxygen, metal ruthenium complexes and metal porphyrin complexes are the most widely used oxygen dyes [13], because ruthenium complexes have broad absorption bands located most often in the blue region (400–480 nm) of the visible spectrum, and they also possess the advantages of moderate brightness, long phosphorescent lifetime, excellent photostability, nontoxicity, and a long Stokes-shift. The enhancement of luminescence and sensitivity of the ruthenium complex for oxygen monitoring has been explored by making use of spin-coated trimethoxysilane (TEOS) xerogel on a gold surface [9]. However, the sensitivity is only improved in the low O_2_ concentration range [12]. On the other hand, [Ru(Phen)_3_]_2_ dyes embedded in mesoporous silica have shown nonlinear calibration plots [13]. Recently, several efforts have been made to investigate mesoporous, micro, and nano-based matrix materials to improve the performance [20,21,22,23,24]. It has been indicated that utilizing micro and nano-materials could enhance the performance of ruthenium complex-based sensors; for instance, improving the limit of detection, stability, and emission intensity [10,25,26]. 

Intensity measurements suffer from many weaknesses and limitations, such as background reflection, drifts of the excitation light sources, inhomogeneous fluorophores dyes [27]. The luminescence life-time-based approach is superior to pure intensity measurements, as it can overcome the main weaknesses of the intensity-measurement method [28]. However, the life-time-based approach requires relatively complex and expensive hardware to control and synchronize the light and camera. One compromise method for intensity measurements and lifetime is the ratiometric method, based on the two different luminophores that exhibit different performance characteristics on varying O_2_ concentration [29,30]. [Ru(bpy)_3_]_2_^+^ as the dye and calcein as the O_2_-insensitive dye were applied to fabricate the ratiometric sensor in [31]. However, the calibration curve was nonlinear, and the sensing characteristics of the sensor need to be further improved in practical application.

The utilization of metallic particles like gold or silver can effectively tune the optical characteristic of fluorophores. The emission spectral properties of [Ru(bpy)_3_]_2_ complexes were examined by doping with silver particles in [32]. Vamsi K studied the effect of different sizes of silver and gold nanoparticles on the fluorescence properties of three kinds of ruthenium complex [33]. In recent years, fibers doped with silver nanoparticles by electrospinning technique was investigated, and the results showed that the sensitivity and linear calibration plots could be tuned by doping with gold or silver particles [34]. However, those reported works of nano-fiber-based sensing films are limited to the lifetime approaches [35]. 

In this paper, a ratiometric sensor based on silver nanoparticles is investigated. Silver nanoparticles (Ag NPs) are doped in nano-porous polymethyl methacrylate (PMMA) to tune the properties of the Ru complex. Coumarin6 is adopted as reference dye to fabricate the ratiometric sensor by electrospinning technique and traditional knifing. The effects of the presence of silver nanoparticles on the sensors are evaluated in terms of calibration curve, sensitivity, precision, response time, and photostability. The performance of the ratiometric sensor is validated for the detection of the oxygen variation of *Chlorella vulgaris*. The results indicate that the ratiometric oxygen sensor can be a cheap and effective tool for real-time applications.

## 2. Experimental

### 2.1. Chemical and Materials

The O_2_-sensitive fluorescent dye Ru(DPP)_3_Cl_2_, Coumarin6, silver nitrate, copper nitrate, chloroform, ethanol, sodium borohydride, and PMMA were purchased from J&K Chemical Company (Shanghai, China) and Aladdin Chemical Company (Shanghai, China); they were all analytical grade. Oxygen and nitrogen gas (99.9% purity) cylinders were provided by Haisheng Company, Qingdao, China.

### 2.2. Synthesis of Silver Nanoparticles

We synthesized the silver nanoparticles according to the reported literature [36]. Briefly, 20 mL of solution containing 2.0 mM AgNO_3_ was added slowly into 60 mL of 2.0 mM NaBH_4_ solution, and the solution was stirred continuously by magnetic stirring apparatus under an ice bath. The basic chemical reaction is shown in Equation (1):

(1) $${\mathrm{AgNO}}_{3}+{\mathrm{NaBH}}_{4}\to \mathrm{Ag}+H_{2}+\frac{1}{2}B_{2}H_{6}+{\mathrm{NaNO}}_{3}$$

### 2.3. Sensor Fabrication

**Figure 1:**
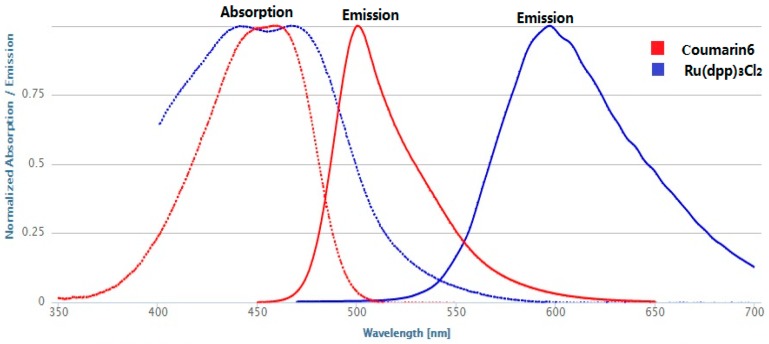
Absorption and emission spectrum of Ru(DPP)_3_Cl_2_ and Coumarin6.

Ru(DPP)_3_Cl_2_ was chosen as the oxygen sensitive indicator. By making use of the Coumarin6 as the antenna dye, the spectrum intensity of the Ru(DPP)_3_Cl_2_ complexes could be improved because the reference fluorescer plays an energy donor role for the indicator. Both the Coumarin6 and the Ru(DPP)_3_Cl_2_ have overlapped absorption spectra band for excitation. As the emission intensity of Coumarin6 is insensitive to O_2_, the characteristic of Coumarin6 allows its use as the reference (Figure 1).

The peak of wave of emission from the Coumarin6 is located at the wavelength of 498 nm (in PMMA), and the Ru(DPP)_3_Cl_2_ emission is located at the peak wavelength of 608 nm (in PMMA). The relatively large difference in the peak of emission ensures the possibility of application due to minimal optical crosstalk. Ratiometric measurements are obtained by simultaneously recording the intensity originated from both dyes. 

The sensor composites were prepared by dissolving 200 mg of PMMA, 1 mg of Coumarin6, 1 mL of metallic nano-silver, and 1 mg of Ru dye in CH_2_Cl_2_:EtOH (*v*/*v*, 9:1) solution. Two methods were used to fabricate the sensing films. One was the traditional method utilizing a drawknife to produce the sensing film on a 125 µm PET (polyethylene terephthalate) foil, and the other approach was to manufacture sensor films by utilizing electrospinning. The handheld electrostatic spinning pump (Bona-Technology Company, Qingdao, China) was used to fabricate electrospun films. In the electrospinning procedure, the solution was prepared by vigorous stirring for 4 h at room temperature (25 °C), and then the prepared stock solution was extracted in a 10 mL plastic syringe equipped with a metallic needle (0.4 mm inner diameter). The voltage of 10 kV was applied, the feed rate of the prepared solution was 0.5 mL/20 min, and the distance was controlled at 8 cm.

### 2.4. Experiment Setup

**Figure 2:**
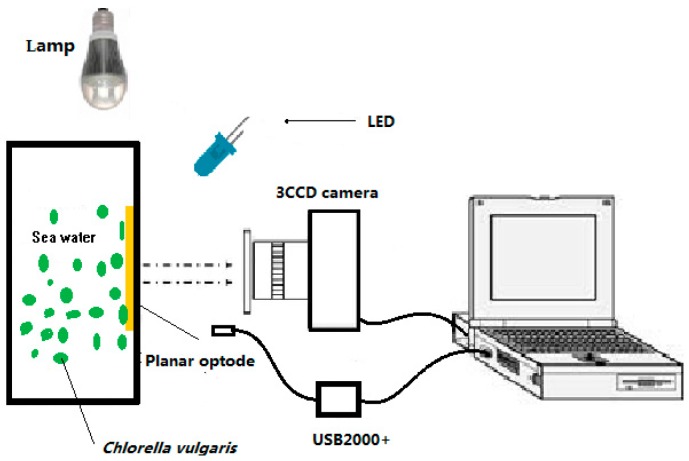
Schematic drawing of the experimental setup. The O_2_ sensor film is based on the O_2_-sensitive luminophore Ru(DPP)_3_Cl_2_. Coumarin6 was used as the reference dye.

The O_2_ measurement setup is illustrated in Figure 2. For the present work, a 450 nm LED (Tianyao companies, Shenzhen, China) was used, and the luminescence measurements of the film were collected by the Ocean Optics spectrometer USB2000+ and JAI AT-200GE 3CCD camera (Daheng Image Company, Beijing, China). Emissions filters which were equipped in front of the 3CCD camera were 470 nm long-pass filters (Nantong Optical glass Company, Beijing, China). The lamp was used to provide light illumination for the *Chlorella vulgaris* in the experiment.

## 3. Results and Discussion

### 3.1. Structure and Morphology of the Sensors

**Figure 3:**
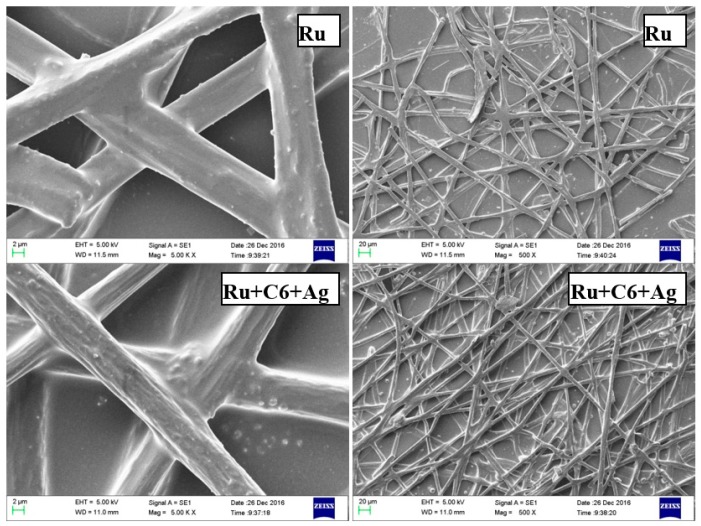
SEM micrographs of the electrospun fiber films based on polymethyl methacrylate (PMMA) at 10 kV under different magnifications. C6: Coumarin6; Ru: Ru(DPP)_3_Cl_2_.

The fiber diameter and morphology of the electrospun film was characterized using scanning electron microscopy (SEM) as shown in Figure 3. It is shown that the 3D network morphology of the sensor in PMMA matrix displays a random fiber orientation which was evenly distributed on the PET foil. The higher surface area of the fibrous-structure by electrospinning ensured faster response to oxygen than traditional films fabricated by knifing. The sensor film showed a consistent fiber diameter, the fibers became thinner after doping with reference Coumarin6, and the average diameter for reference-free sensors and reference-containing sensors were 8 µm and 10 µm, respectively. 

### 3.2. Calibration of the Oxygen Sensor Film

**Figure 4:**
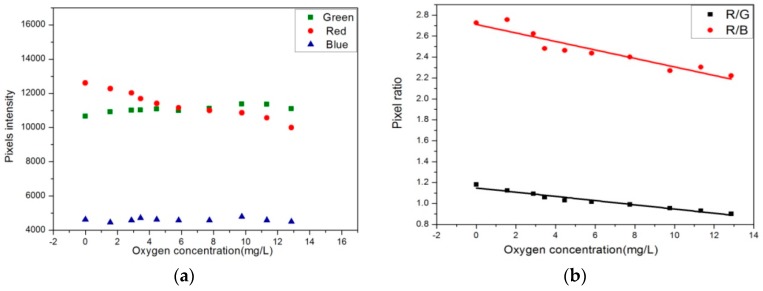
(**a**) Pixel intensity of the red (●), green (■), and blue (▲) image for the Ag-containing film at different O_2_ concentrations by 3CCD camera; (**b**) Pixel ratio values for the O_2_ sensor film in 30‰ artificial seawater at 17 °C.

The emission intensity was recorded by the corresponding three spectrum channels of a 3CCD camera. The pixel intensity of the green channel and the red channel were dominated by the luminescence from the Coumarin6 and the Ru(DPP)_3_Cl_2_, respectively, while the blue channel represented the blue LED excited light. As mentioned above, the Coumarin6 served as an internal reference insensitive to O_2_. It is shown in Figure 4a that the emission from the blue LED (blue channel) could not be fully absorbed by the sensor and matrix; the residual blue LED light was unaffected by O_2_ concentration and could also be utilized as a reference signal, like the luminescence from Coumarin6 (green channel). The ratio of average pixel light intensity of the green channel to the red one was used for oxygen measurement. The R-squared values of the two curves for the G and B channels were calculated, and linear fit values were 0.994 and 0.883, respectively (Figure 4b). These results confirmed that the Coumarin6 is suitable as reference luminescence for ratio measurement.

Artificial sea water at a concentration of 30‰ was prepared for the calibration process. The dissolved oxygen concentration was continuously measured by O_2_ microelectrode (Unisense O_2_ Microsensor, Denmark). Because luminescence of the sensor was affected by temperature and salinity, the calibration process was carried out at the same temperature (17.0 ± 0.2 °C). According to Stern–Volmer theory, the relationship of fluorescence intensity, lifetime change, and oxygen concentration is expressed as in Equation (2) [37]:

(2) $$I_{0}/I=\tau_{0}/\tau =1+K_{\mathrm{sv}}\left[O_{2}\right]$$

where τ and I, respectively, are the luminescence lifetime and intensity at an O_2_ concentration of, while τ_0_ and I_0_ are at O_2_ free condition. K_sv_ indicates the Stern–Volmer constant.

**Figure 5:**
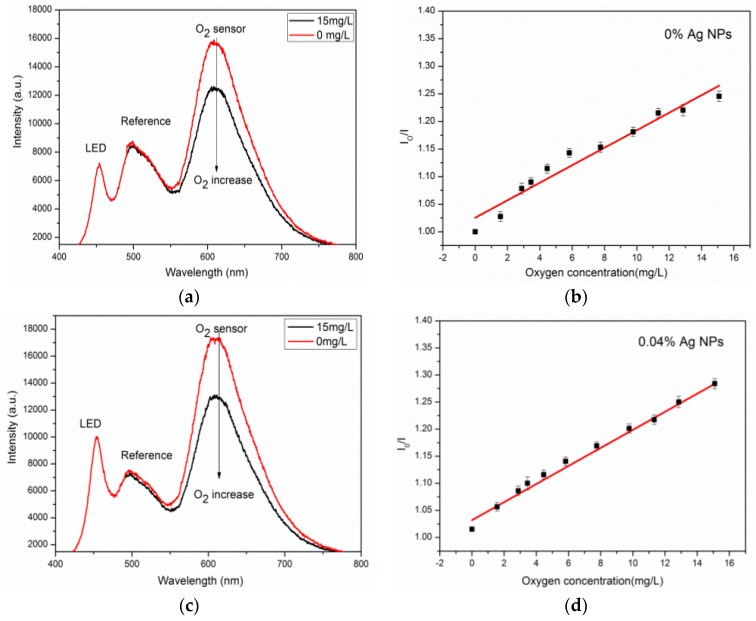
(**a**) Emission spectra of the Ag-free film; and (**c**) Ag-containing film by electrospinning upon exposure to the oxygen concentrations of 0–15 mg/L; (**b**,**d**) Stern–Volmer plot derived from quenching-based data of the sensor film. Ag NP: Silver nanoparticle.

The Stern–Volmer plots of Ru(DPP)_3_Cl_2_ in PMMA matrices for oxygen concentrations in the range of 0.0–15 mg/L were evaluated. K_sv_ values of 0.015 and 0.019 for Ag-free and Ag NPs-doped, respectively, were obtained as seen in Figure 5b,d. Note that the Ag-free film showed a non-linear curve for the Stern–Volmer plots (R^2^ = 0.9503). From Figure 5, the plots of the film doped with Ag revealed good linearity (R^2^ = 0.9837). This improvement of linearity is suggested to be a result of the features of silver NPs.

We investigated the intensity of the Ru(DPP)_3_Cl_2_ indicator both in the absence and the presence of the metallic silver with the illuminating effect of Ag NPs. The Ag-containing sensor film exhibited increased intensity by 1.2 times (the concentration of oxygen was 0 mg/L) in PMMA matrices compared to the Ag-free sensor film. As shown in Figure 5b, the linear dynamic response ranges only covered the scope between 0 and 6 mg/L However, for the Ag NPs-doped sensor, it was observed that the calibration curve became linear and the response ranges were enhanced for 0–15 mg/L. This is due to the spectral consequences considering absorption characteristics of the Ag NPs [34], and the function of Ru(DPP)_3_Cl_2_ undergoing a metal-to-ligand charge-transfer (MLCT) electronic transition. The emission from the Ru(DPP)_3_Cl_2_ was located at a peak wavelength of 608 nm (in PMMA), which overlaps with the absorption of Ag NPs. Therefore, within the same PMMA matrix, the emission of Ru(DPP)_3_Cl_2_ may be absorbed by the Ag NPs, resulting in an energy transfer to tune the sharp response of the indicator for the range between 0 and 6 mg/L.

**Figure 6:**
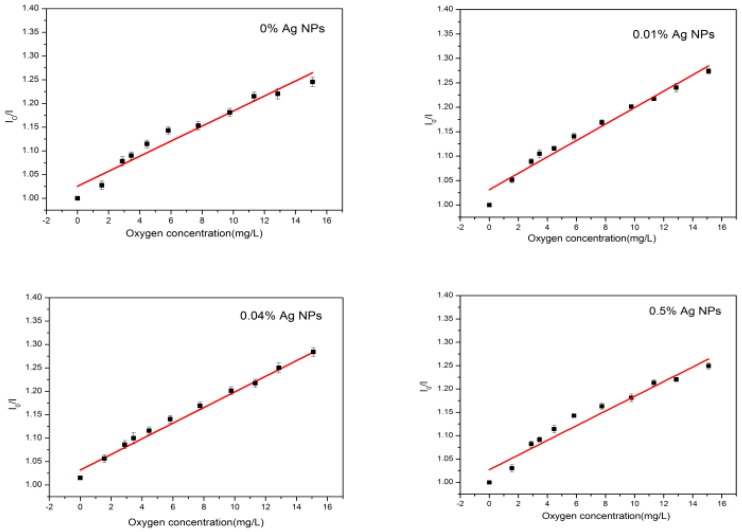
Effects of the calibrating plot with different Ag NPs concentration.

Figure 6 shows the effect on the calibration plot with an increase in Ag NPs concentration. For optimized concentration of the Ag NPs (0.02 wt % and 0.04 wt %), it is obvious that Ag NPs significantly affect the quenched property—the sharp slope at the low oxygen range becomes tuned and linear.

Fabrication of electrospun and knifing of films with different Ag NPs concentrations was conducted. However, with excess of silver, the effect of the Ag NPs on the oxygen indicator Ru(DPP)_3_Cl_2_ vanished, and the slope at the low oxygen range 0–6 mg/L became sharp again. On the other hand, concentrations higher than 0.5 wt % resulted in an inhomogeneous film due to the aggregation of the Ag NPs. With increasing Ag NPs concentrations, the aggregation of the nano-particles comes into force, thereby causing formation of larger particles, and hence leading to changes in the physical and chemical properties of Ag NPs depending on the size and shape. 

### 3.3. Oxygen Sensitivity and Accuracy Evaluation

**Figure 7:**
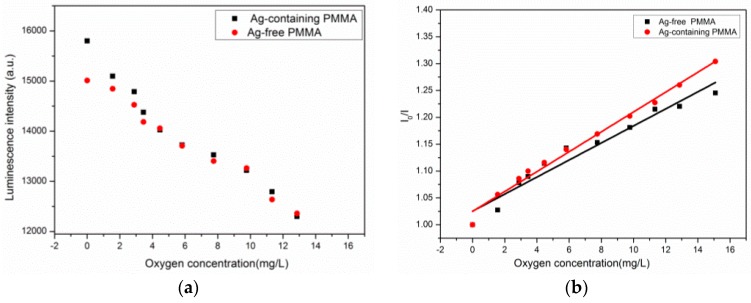
(**a**) Luminescence intensity; and (**b**) Stern-Volmer plot obtained from the two films by electrospinning in seawater. Black and Red represent Ag-free and Ag-containing film, respectively.

In the present study, the cross-sensitivity and accuracy of the sensors were evaluated to examine the characteristics of the sensor film. O_2_ can generally be considered the only major quencher due to the specificity of quenching processes in aquatic environments. Although SO_2_ gases and nitrogen oxides show an interference for PtOEP (platinum-octaethyl-porphyrin) [38] and Ru(DPP)_3_Cl_2_ [39], the existence of these gases can be neglected. Utilizing the O_2_ measurements systems, the luminescence intensity and the I_0_/I value of the two sensing films were quantified and are illustrated in Figure 7. The luminescence intensity values could reflect the dynamic response working range of O_2_ and the K_sv_ value of the two films (Figure 7; Table 1). 

When the O_2_ concentration increased from 0 to 13 mg/L, the acquisition luminescence intensity (arb.unit) for the Ag-free film decreased from an average of 15,023 to 12,455, and the luminescence intensity of the Ag-containing film decreased from 15,915 to 12,397. The slope of the Ag-containing films was higher than that of Ag-free films, and the nano-silver particles exhibited a change in sensitivity—the K_sv_ was tuned to be 0.019 from 0.015 (about 26.66% enhancement). 

**Figure 8:**
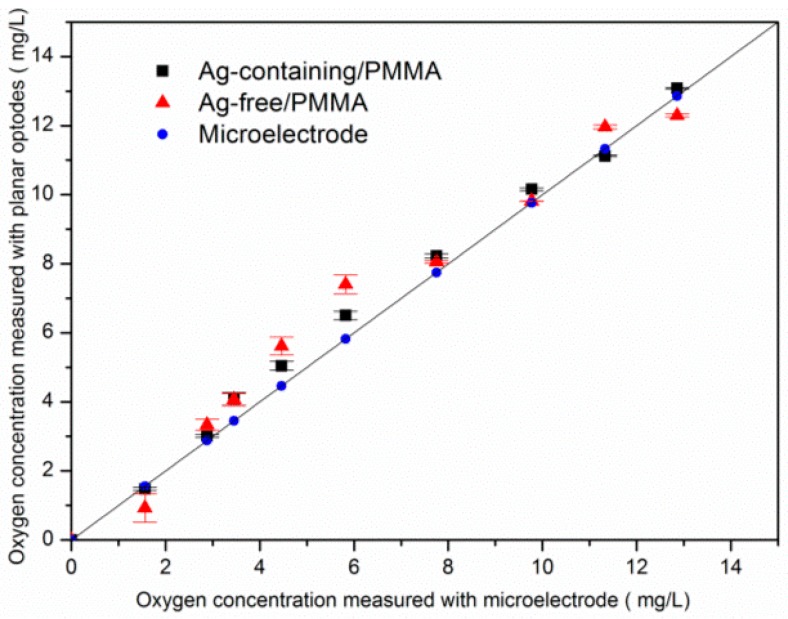
O_2_ concentrations measured in seawater with microelectrode (round) versus calibrated recordings of the two films: Ag-free film (triangle), and Ag-containing film (square).

Figure 8 and Table 2 illustrate the error, analyzing between ratiometric films and the results measured by an O_2_ microelectrode. The average concentration of dissolved oxygen measured by the Ag-containing film showed superior linear relationships to the Ag-free film. 

**Figure 9:**
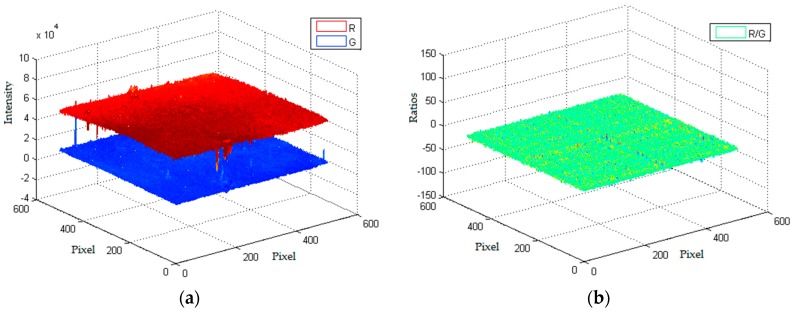
Surface plots of (**a**) intensity images and (**b**) image ratios of Ag-containing film at 13 mg/L.

The improvements of the film doping with Ag NPs in measurement accuracy have been illustrated with error bars. The error bars of the Ag-free film are generally larger than the Ag-containing film for the concentration range of 0–13 mg/L. It is noted that the Ag-containing film possessed superior behavior in O_2_ depleted and supersaturated conditions. As shown in Table 2, the added Ag NPs could tune the performance of the sensing film and make the sensors have better linearity compared to the film without additives. Figure 9 shows the 3D plots of two channel intensity output and ratio results at 13 mg/L. There was some noise signal caused by inhomogeneous distribution of the indicator dye and un-uniform excitation light (Figure 9). However, after the ratio process, the effects of interference can be significantly reduced. Table 3 illustrate the previous studies about the intensity and ratiometric oxygen sensors of metal Ru compounds.

### 3.4. Characteristics of Detection and Resolution 

In the present study, we measured the fluorescence intensity of the sensor film for the concentrations 0 and 5.82 mg/L every 5 min, a total of 10 times, and the standard deviation of the ratio of the fluorescence intensity was calculated. At a signal-to-noise ratio of 3, the minimum limit of detection (LOD) was calculated by Equation (3):

(3) $$\mathrm{LOD}=3\times S/K_{\mathrm{sv}}\left[O_{2}\right]$$

where LOD and S are the minimum limit of detection and the standard deviation of the ratio, respectively, K_sv_ indicates the Stern–Volmer constant. The LOD values were 0.182 mg/L and 0.160 mg/L for Ag-free film and Ag-containing film, respectively.

In order to evaluate the resolution of the sensors, we changed the dissolved oxygen concentration in the calibration solution slowly and recorded the change in the fluorescence intensity of the sensing membrane. The resolution of the Ag-free film and the Ag-containing film were 0.21 mg/L and 0.24 mg/L, respectively.

### 3.5. Long Term Stability 

**Figure 10:**
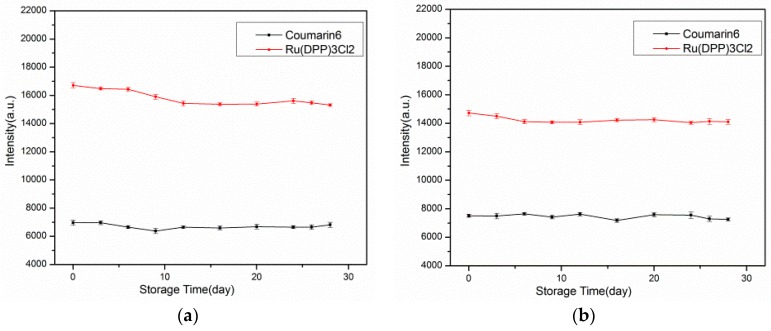
Effects of storage time on the (**a**) Ag-free film; and (**b**) Ag-containing film fabricated by electrospinning.

The stability over time is a key analytical figure of merit for oxygen sensors. An experiment was conducted to evaluate the proposed sensing films by putting the films in sea-water at 25 °C for a period of 4 weeks. The test results for both films are shown in Figure 10. 

Because of leaking of the indicator in water, the average intensity of the Ag-free film showed decreases of 3.19% and 8.38% for Ru(DPP)_3_Cl_2_ and Coumarin6, respectively, after 28 days. The fluorescence intensity of the Ag-containing film showed decreases of 3.33% and 5.08% for Ru(DPP)_3_Cl_2_ and Coumarin6, respectively. The Coumarin6 showed better stability than the oxygen indicator, owing to the good hydrophobic property of Coumarin6.

### 3.6. Effect of pH and Metal Ions 

**Figure 11:**
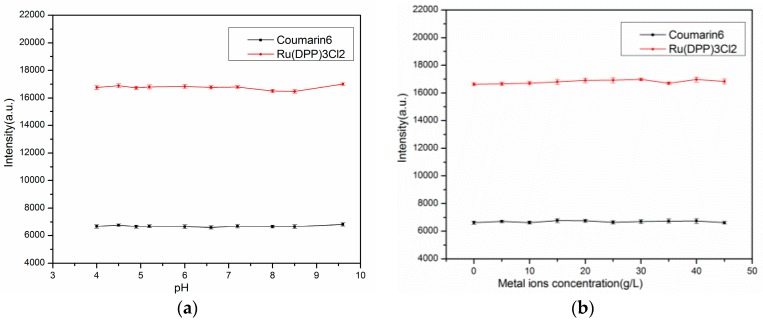
Effects of (**a**) pH; and (**b**) metal ions on the Ag-containing sensor by electrospinning.

It is well known that the some metal ions are ionic quenchers; for instance, bromide. Sulfide can ‘‘quench’’ by undergoing a chemical addition reaction with the fluorophore [43]. To evaluate the robustness of the proposed sensing films, an interferences test was performed by changing the pH values of the calibration solution from 4 to 10. Figure 11 shows the effects of varying pH values on the intensity of both Ru(DPP)_3_Cl_2_ and Coumarin6. Finally, a metal ions solution was prepared by mixing silver nitrate and copper nitrate (with a weight ratio of 1:1) in an aqueous solution, and the solution was added into the calibration solution. It was observed that the increasing concentrations of metal ions (Cu^2+^ and Ag^+^) did not affect the intensity of Ru(DPP)_3_Cl_2_ and Coumarin6 (Figure 11b). Therefore, these interference effects on the sensing performance can be considered negligible, and it is possibly due to the characteristics of the ion-impermeable matrix material PMMA.

### 3.7. Response Time of Ru(DPP)_3_Cl_2_

**Figure 12:**
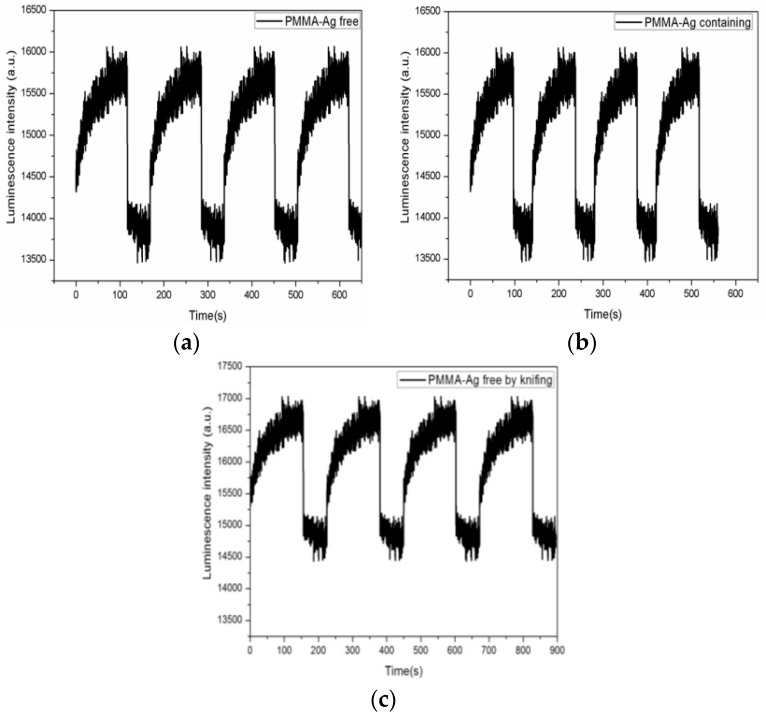
Response time and fluorescence intensity change of the (**a**) Ag-free film; (**b**) Ag-containing film by electrospinning; and (**c**) Ag-free film by knifing upon switching between air saturation and fully deoxygenated.

In real-time applications, rapid response is a critical performance factor in, for instance, industrial process monitoring and biological measurements. Generally, the response times of optical oxygen sensors are defined as the 90% response and recovery times when switching alternately between 100% oxygen and 100% nitrogen condition, respectively. Figure 12 shows the dynamic response of the films when switching between fully oxygenated and fully deoxygenated condition, respectively. 

The response times of the Ag-free film and Ag-containing film by electrospinning were 1.5 s and 1.0 s upon switching from deoxygenated to air saturation and 65 s and 45 s from air saturation to fully deoxygenated, while the response time of the Ag-free film by knifing was 2.0 s upon switching from deoxygenated to air saturation and 104 s from air saturation to fully deoxygenated. By utilizing the electrospinning, the mesoporous structures exhibited noticeable improvements in terms of enhanced response times. For the PMMA-based sensor, the response time of the silver-containing film was faster compared to the Ag-free sensor film. The signal changes were fully reversible.

### 3.8. Photostability

**Figure 13:**
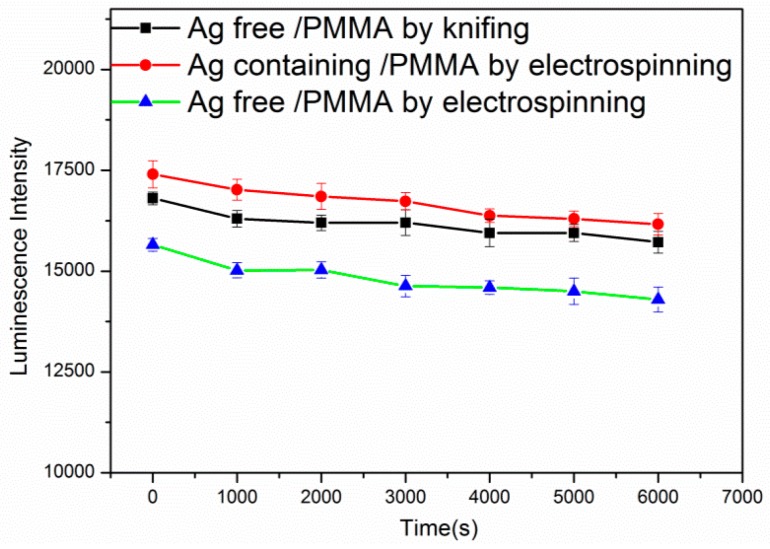
Photostability of the Ag-free film, Ag-containing film by electrospinning, and the Ag-free film by knifing.

All fabricated sensing films were continuously illuminated at 450 nm for about 2 h. The photostability of the three sensing films with Ru(DPP)_3_Cl_2_ was tested. Figure 13 shows that the photostability of the three sensing films changed slightly. The luminescence intensity was decreased by 8.67% for the Ag-free film by electrospinning, by 7.10% for the Ag-containing film by electrospinning, and decreased by 7.34% for the Ag-free film by knifing after the 2 h luminescence. 

## 4. Application of the Optical Sensors for Oxygen Measurements of *Chlorella Vulgaris*

### 4.1. Chlorella Vulgaris Samples

*Chlorella vulgaris* was obtained from the Ocean University of China and was grown phototrophically in liquid BG11 medium at (25 ± 1) °C under 250 µmol photons s^−1^·m^−2^, with a 12 h:12 h light:dark ratio. To validate the ratiometric sensing system for real-time application, an experiment was conducted to measure oxygen variations for photosynthesis and respiration process of *Chlorella vulgaris* for 2 days. A quartz glass tank was sealed with a transparent cap to prevent CO_2_ and O_2_ exchanges with air during the measuring experiment.

### 4.2. Imaging of the Two-Dimensional O_2_ Distribution for Chlorella vulgaris 

**Figure 14:**
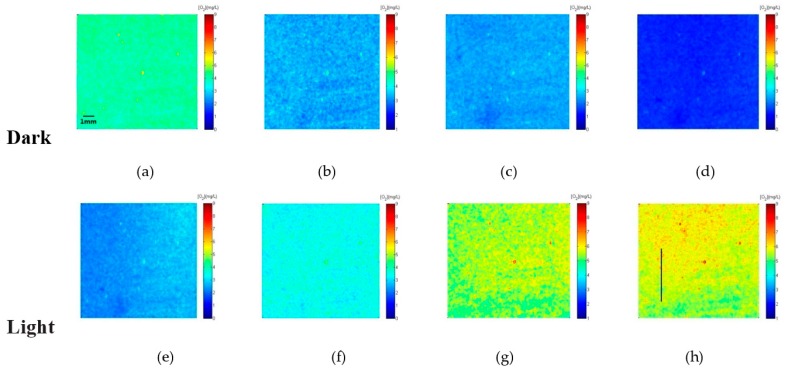
Time series (5 min intervals) recording of the oxygen distribution. The positions of the profiles presented in Figure 15 are the marked line in Figure 14h. (**a**–**d**) is the dark condition, (**e**–**h**) is the light condition.

By applying the presented ratiometric Ag-containing film, the two-dimensional O_2_ distribution caused by *Chlorella vulgaris* with different photosynthetic and respiration performance were measured. Results in Figure 14 show the measurements of O_2_ for *Chlorella vulgaris* in the dark (Figure 14a–d) and in the light irradiation (light at 250 µmol photons s^−1^·m^−2^) (Figure 14e–h). The scale of concentration of dissolved oxygen is expressed with a color bar. 

During the light cycle, *Chlorella vulgaris* released oxygen by photosynthesis, and this activity caused the oxygen concentration to increase. The maximum values of 7.2 mg/L after 65 min light exposure was observed. Within the experimental period, the concentration of the dissolved oxygen was varied from 1.1 mg/L to 7.2 mg/L. When the light was turned off to simulate night condition, the dissolved oxygen distribution was gradually decreased to a minimum value of 1.1 mg/L after 15 min. These variations of O_2_ concentration reflect the of photosynthesis and respiration activities of *Chlorella vulgaris*. 

**Figure 15:**
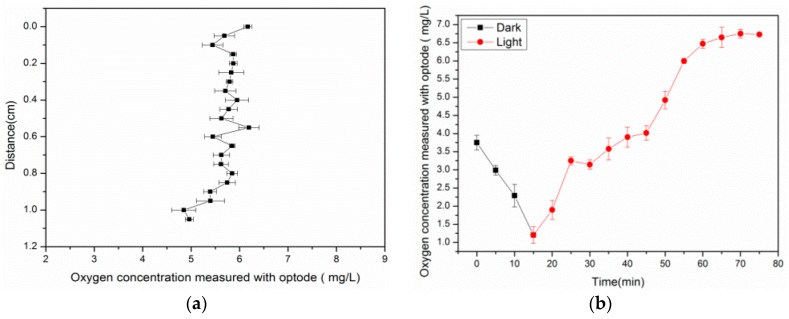
(**a**) Concentration profiles extracted from Figure 14h; (**b**) Mean O_2_ concentration measured with Ag-containing film.

The vertical profiles of the dissolved oxygen (Figure 15a) were extracted from Figure 14h (corresponds to the vertical line). It was shown the surface oxygen was higher than at 1 cm below the surface. This means that *Chlorella vulgaris* on the surface received more light and had strong photosynthesis, as the light was attenuated with increasing depth. The photosynthesis process of *Chlorella vulgaris* was not active. The experimental results demonstrated that the sensor film was a valuable tool for resolving the dissolved oxygen distribution at a spatiotemporal resolution.

## 5. Conclusions

In this work, ratiometric oxygen sensor films were fabricated with the combination of Ru(DPP)_3_Cl_2_ and Coumarin6 in PMMA matrices doped with Ag NPs. Different methods of membrane preparation were used—namely, knifing and electrospinning. The ratio measurement of Ru(DPP)_3_Cl_2_ complex and Coumarin6 reference was achieved with 3CCD camera. It was demonstrated that a 26.66% enhancement of K_sv_ was achieved by doping with Ag NPs. The linear working range was improved from 0.0–6 mg/L to 0.0–15 mg/L for the PMMA matrix. The performance of the ratiometric films were evaluated, and the results indicated that the nanofiber film doped with Ag NPs had good stability, response time, and photostability. The interferences of pH and metal ions on the film were investigated; the results showed limited effects on the film, and can be considered negligible. The application of using the nanofiber Ag-containing film to measure the photosynthesis and respiration activity of *Chlorella vulgaris* is presented, and the results showed that the ratiometric sensor film is applicable for biological applications in aqueous conditions.

## Figures and Tables

**Table 1:** K_sv_ and standard error values of the sensor by electrospinning.

Film Type	K_sv_ (mg/L^−1^)	Standard Error	R^2^
Ru(DPP)_3_Cl_2_ in PMMA	0.015	0.00114	0.9503
Ru(DPP)_3_Cl_2_/Ag in PMMA	0.019	0.00072	0.9837

**Table 2:** Comparison of fabricated Ag-free, Ag-containing film, and electrodes for O_2_ measurements. RE: relative error.

Unisense O_2_ Electrodes	Ru(DPP)_3_Cl_2_			
	PMMA/Ag NPs-Free		PMMA/Ag NPs	
Measured	Calculated	RE	Calculated	RE
0	0	0%	0	0%
2.88	3.33	15.87%	3.00	4.51%
5.82	7.40	27.25%	6.51	11.8%
75	8.06	4.00%	8.22	6.15%
9.77	9.81	0.48%	10.16	4.00%
11.33	11.96	5.60%	11.12	1.83%
12.86	12.29	4.37%	13.08	1.75%

**Table 3:** Luminescence properties of oxygen immobilized in polymer matrix. λ exc/em is spectral peaks of excitation and emission; SVP means Stern–Volmer plot; I_R_ = I_0_/I_100_ is the ratio of fluorescence intensity at 0% O_2_ and 100% O_2_; QD is Quantum Dot; PEG is poly ethylene glycol.

Dye/Matrix	Method	Dope	λ exc/em	I_R_	Comments	Ref.
[Ru(bpy)_3_]^2+^ in sol–gel matrix	Intensity	-	470/680	~10.6	Good linear SVPs only at low oxygen; fast response 5 s, 10 s; no leaching effect; good photostable.	[35]
[Ru(bpy)_3_]^2+^/Calcein	Ratio	-	423/515,627	~1.8	Ratiometric sensing method; non-linear SVPs; good antijamming capability.	[31]
Ru(bipy)_3_^2+^ in EC matrix	Intensity	Ag NPs	460/610	~2.0	Porous structure; good linear SVPs; intensity method; good mechanical strength.	[34]
[Ru(bpy)_3_]^2+^ + CdSe–ZnS QD in sol–gel matrix	Ratio	-	470/520,600	-	Stable self-referenced oxygen sensor; suitable for long term use; independent of fluctuations in excitation; QDs are temperature dependent.	[40]
Ru(DPP)_3_Cl_2_ + Oregon Green in sol–gel nanoparticles	Ratio	-	488/610	~6	Particles size 50–300 nm; PEG added as a steric stabilizer; also good for sensing in solution; stable to leaching and decomposition; reference dye Oregon Green is pH sensitive; response time below 1 s.	[41]
Ru(phen)_3_ + NBD-PE in polymerized phospholipid vesicle	Ratio	-	450/510,600	~3	Chemically-stabilized phospholipid vesicle sensors; general linear response over the entire range of dissolved O_2_ encountered in biological systems; nanometer-sized; biocompatible chemical sensors.	[42]
Ru(DPP)_3_Cl_2_ + Coumarin6 in PMMA matrix	Ratio	Ag NPs	450/498,608	~1.5	Fibrous structure by PMMA; good linear SVPs; ratiometric approach show good antijamming capability; general response 1.0 s, 45 s; good mechanical strength.	This study
